# Numerical Simulation of Temperature Distribution during Mild Moxibustion

**DOI:** 10.1155/2022/6604372

**Published:** 2022-06-16

**Authors:** Honghua Liu, Zhiliang Huang, Lei Wei, He Huang, Qian Li, Han Peng, Mailan Liu

**Affiliations:** ^1^Hunan University of Chinese Medicine, Changsha 410208, China; ^2^Hunan City University, Yiyang 413000, China; ^3^Hunan Institute of Science and Technology, Yueyang 414006, China

## Abstract

Mild moxibustion is a treatment approach belonging to moxa-hanging moxibustion. The burning end of the moxa stick is kept at a fixed distance from the moxibustion skin, aiming to make the patient feel warm without burning pain. The appropriate temperature distribution is critical for the mild moxibustion treatment. The purpose of this paper is to improve the efficacy of mild moxibustion on human tissues. By combining the radiative and conductive models with surface-to-surface heat transfer, biological heat transfer simulations are realized based on biological tissues in particular media. A finite element model of mild moxibustion was established to obtain the characteristics of skin tissue temperature distribution under various conditions. The model considers multiple factors, such as the moxa-burning temperature, the stick-to-skin distance, the moxa stick sizes, and the ambient temperature. The results show that the temperature distribution under various conditions is centered at the moxibustion point and the temperature decreases in the surrounding direction. The higher the moxa-burning temperature, the higher the skin surface temperature and the worse the stability in heating. The stick-to-skin distance is inversely proportional to the skin surface temperature. The moxa stick diameter is proportional to the skin surface temperature. The longer the moxibustion time, the higher the skin surface temperature. And the temperature change gradually flattened in the late stage of mild moxibustion. Finally, a set of moxibustion conditions with optimal temperature distribution was obtained by comparing the data of all groups.

## 1. Introduction

Moxibustion is one of the most widely used thermal therapies in Chinese medicine [1] and is a traditional medicine therapy using moxa or mugwort herb ground up to fluff that burned directly on an acupuncture point, which is called direct moxibustion, or burned indirectly one inch or two away from the skin, which is called indirect moxibustion [2]. Especially, systematic reviews [3, 4] have demonstrated the effectiveness of moxibustion for correcting breech presentation and indicated that moxibustion is more effective than medication in ulcerative colitis. Possible explanations for the effectiveness of moxibustion are thermal effects, nonthermal radiation effects, and pharmacological effects (excerpts from tobacco and wormwood) [5]. The moxibustion can enhance the body's function to prevent and treat diseases [6–11]. By heating specific acupoints or areas on the body surface with ignited substances, the functions of meridians and organs are regulated to treat diseases [12, 13]. Li et al. present the initial temperature of four tissues, such as nonacupuncture point, acupuncture point, healthy skin, and tumor skin, and then analyzed temperature distribution of them subject to moxibustion, and at last gave the conclusion that the moxibustion obtains therapeutic effectiveness through heat infiltration [14]. The key indicator of thermal stimulation is temperature [15]. When the stimulation temperature of moxa-heat moxibustion is between 46°C and 48°C, the patient's discomfort and the damage caused by moxa-heat moxibustion are minimized [16]. When the diameter of the moxibustion stimulation area is 2.0 cm∼3.0 cm, an excellent therapeutic effect can be achieved [16]. The skin stimulation adopts about 45°C, and the muscle stimulation adopts about 42.5°C. In this way, an analgesic effect can be produced [16]. To ensure the thermal penetration of mild moxibustion, it is necessary to ensure that the muscle temperature reaches 42.5°C. The therapeutic effect of moxibustion is reflected in the temperature distribution of the acupoints or lesions where mild moxibustion is applied. Scholars studied the temperature distribution during mild moxibustion and drew some conclusions. Wang et al. paid attention to what effects may be produced by the thermal stimulation at two temperatures below and above 43°C on cardiac function in rates, as well as on skin in the local site of moxibustion [17]. Yi et al. discussed the influences of the moxibustion on the Bonghan ducts and Bonghan corpuscle in detail [18]. Lin et al. investigated the changes in skin surface temperature at the SP6 acupuncture point in order to develop a reference point for clinical use [19]. Noh et al. observed blood perfusion as well as the skin temperature of the applied region after moxibustion [20]. Cheng et al. employed an infrared thermal imager to monitor the temperature field of the skin tissue after mild moxibustion and compared the temperature distribution under various moxibustion methods [21]. Limited by the experimental cost and time, the temperature distribution of few experiments was monitored. Li et al investigated the changes of skin temperature under different stimulation points and repeated moxibustion stimulation [22]. Ying et al. explored the thermal effects of various moxibustion methods (e.g., mild moxibustion, aconite moxibustion, and ginger moxibustion) on the Zusanli acupoint by observing the temperature curve of the acupoint surface [23]. Li et al. performed the simulation of skin temperature distribution during mild moxibustion based on experiments [24], but they did not consider the influence of various factors on the skin temperature distribution during mild moxibustion. Ben and Xia simulated the temperature distribution of the skin tissue [15, 25]. The former simulation used a convective heat transfer model, which was inconsistent with moxibustion and mainly relied on radiative heat transfer. The latter modeled the mild moxibustion based on radiative heat transfer and revealed the influence of some parameters on the efficacy of mild moxibustion. However, he did not systematically analyze the selected parameters and did not consider the differences in the burning temperature of moxa sticks from different origins. Xu et al. studied the local skin temperature to varying stick-to-skin distances and completed a univariate analysis of the distance [26]. Shen et al. determined whether traditional moxibustion produces a unique infrared radiation spectrum compared to that of nonspecific controls and examined the relationship between these spectra and that of the surface of the body at an acupuncture point [27]. Solovchuk et al. used magnetic resonance imaging to measure the skin temperature during moxibustion [28]. Comparison with numerical simulation results confirmed that radiative heat transfer plays a crucial role in moxibustion therapy. Song et al. discussed the relationship between spectra characteristics and the effect of moxibustion [29]. Sun et al. used an infrared thermal imaging camera to study the thermal performance of burnt moxa sticks with and without ash removal [30]. They compared the temperature distribution of pig abdominal skin in two conditions based on experiments.

The above studies analyzed the skin surface temperature distribution during mild moxibustion through simulation and experiment methods, which is helpful to improve the effect of moxibustion. However, limited by experimental methods and computational efficiency, the above studies ignored the temperature distribution inside the skin. As a critical indicator of mild moxibustion, temperature penetration has a significant impact on the therapeutic effect. Therefore, it is necessary to establish a reliable mathematical model to analyze the three-dimensional temperature field inside the skin during moxibustion. To improve the therapeutic effect of mild moxibustion, the influence of crucial moxibustion parameters on the temperature distribution must be quantitatively analyzed and optimized accurately. The parameters contain the moxa-burning temperature, the moxa stick sizes, the ambient temperature, etc.

In this paper, Section 2 establishes a three-dimensional heat transfer model, introduces the theoretical models of biological heat transfer and radiation heat transfer, sets up convenient parameters for simulation, and verifies the simulation model through experiments. In Section 3, the model in standard condition is calculated, and the basic characteristics of the temperature field are obtained. Then, a univariate analysis was performed for each influencing factor. The experimental design analyzes the effect of these parameters on thermal penetration.  Section 4 draws some conclusions.

## 2. Methods

Because COMSOL has powerful multiphysics simulation capabilities, this paper uses this software for simulation. In the COMSOL software, we built a reliable physical model to obtain an accurate 3D temperature field inside the skin. The model can simulate the moxibustion process in a three-dimensional coordinate system and obtain relatively accurate results. Therefore, this model can be used to optimize the temperature distribution inside the skin to improve the effect of gentle moxibustion.  Section 2.1 creates a 3D physical model for moxibustion.  Section 2.2 established a reliable mathematical model of moxibustion.  Section 2.3 defines the boundary conditions for the moxibustion simulation.  Section 2.4 verifies the correctness of the model through experiments.

### 2.1. Physical Modeling

This paper builds multi-layer physical models in COMSOL to mimic the morphology of real skin tissue. The human skin essentially consists of three layers, namely the epidermis, dermis, and subcutaneous tissue or hypodermis [31]. However, because there is no distinction between epidermis and dermis in biological models, epidermis and dermis are collectively referred to as skin tissue in modeling. The biological tissue in the proposed simulation model was defined as three layers, including the first layer of skin tissue, the second layer of fat, and the third layer of muscle. The thicknesses of the three tissue layers were set as 2.2 mm, 12.4 mm, and 10.4 mm, respectively. The material parameters of biological tissues were extracted from the material library of COMSOL, as listed in Table 1.

**Figure 1:**
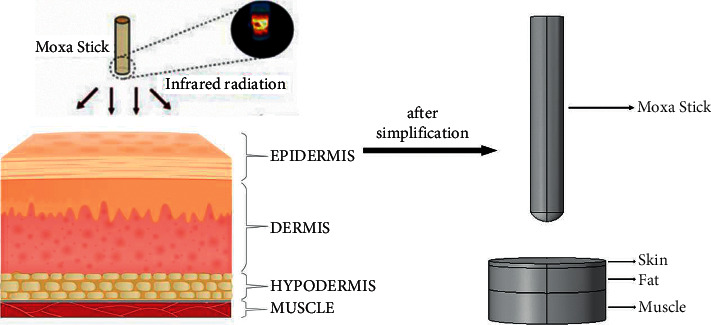
The physical model of mild moxibustion.

After the skin tissue is established, a moxa stick model is established above the skin tissue. The structure of the moxa stick burning end was modeled as part of a sphere. Since the influence of natural convection in the air was ignored, convective heat transfer at two places is not considered: ① the influence of the upper part of the moxa stick on the skin temperature distribution was negligible, and ② the air convective heat transfer between the moxa stick burning end and the skin was negligible. Consequently, the proposed model ignores the air area above the skin surface. To maintain the model's integrity, it shows the nonburning cylindrical portion of moxa sticks, as shown in Figure 1.

**Figure 2:**
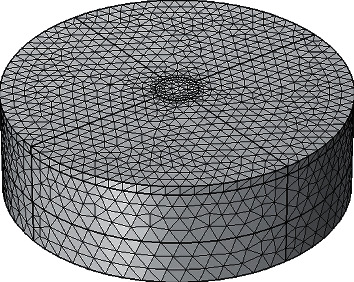
The meshing on biological tissue.

The average temperature of a circular area centered on the moxibustion point was used to compare the simulation and experimental results. To ensure accuracy, the skin surface area with a radius of 5 mm centered on the moxibustion point was divided into a denser grid, as shown in Figure 2.

### 2.2. Mathematical Modeling

The heat transfer from the moxa stick burning end to the biological tissue mainly depends on the surface-to-surface radiative heat transfer. Then, the heat absorbed on the skin by thermal radiation becomes a heat source inside the biological tissue. It is the basis for heat transfer analysis of biological tissues, resulting in a temperature field distribution throughout the treated tissue. Therefore, two modules of radiation heat transfer and solid heat transfer are selected in the COMSOL physical section. In the solid heat transfer module, biological tissue in a special medium is selected to convert solid heat transfer into biological heat transfer.

The surface-to-surface radiative heat transfer is expressed as

(1) $$\begin{array}{c} \left\{\begin{array}{c} J=\varepsilon e_{b}\left(T\right)+\rho_{d}G,\\ e_{b}\left(T\right)=n^{2}\sigma T^{4}, \end{array}\right. \end{array}$$

where *J* denotes the effective radiation; *G* represents the input radiation; *e*_*b*_(*T*) is the radiative heat flux density of the blackbody as a function of temperature; *ρ*_*d*_ is the surface reflectance; *T* denotes the temperature; *σ* is the blackbody radiation constant, i.e., (5.67 × 10^−8^Wm^−2^K^−4^); and *n* is the refractive index of the transparent medium (*n* = 1).

The heat conduction simulation in biological tissue is solved by the Pennes formula [32], written as

(2) $$\begin{array}{c} \rho c\frac{\partial T}{\partial t}=\nabla \left(k\nabla T\right)+\omega_{b}C_{b}\left(T_{b}-T\right)+q_{m}+q_{r}, \end{array}$$

where *T*, *ρ*, *c*, and *k* denote the temperature, density, specific heat, and thermal conductivity of the tissue, respectively; *ω*_*b*_ represents the blood perfusion rate; *C*_*b*_ and *T*_*b*_ are the specific heat and temperature of the blood; *q*_*m*_ is the heat production rate of the tissue metabolic; and *q*_*r*_ denotes the external heat source.

### 2.3. Moxa Burning Curve Settings

The moxa-burning curve was defined based on the burning experiment results. The measured burning temperature of moxa sticks was used as the input parameters of the simulation model.

Although the maximum combustion temperature of moxa sticks produced by various manufacturers is different, the temperature change law is similar. The burning moxa sticks initially rise in temperature until the temperature reaches a maximum. During moxibustion, the burning area of moxa sticks will continuously increase the radiation as the burning increases. However, as the intensification of the combustion, the moxa ash has covered the end of the moxa stick burning area gradually. The temperature of the moxa stick burning end gradually decreases until the end of the mild moxibustion. During the process, the moxa ash acts as a barrier to thermal radiation. Therefore, simple harmonics were employed to simulate the temperature change in the actual situation of gradually accumulating dust. The simulation model is expressed as

(3) $$\begin{array}{c} T_{a}=400+200\text{cos}\left(\frac{2\pi }{500}t\right), \end{array}$$

where *T*_*a*_ is the moxa stick burning temperature (°C), and *t* represents the time (s).

**Figure 3:**
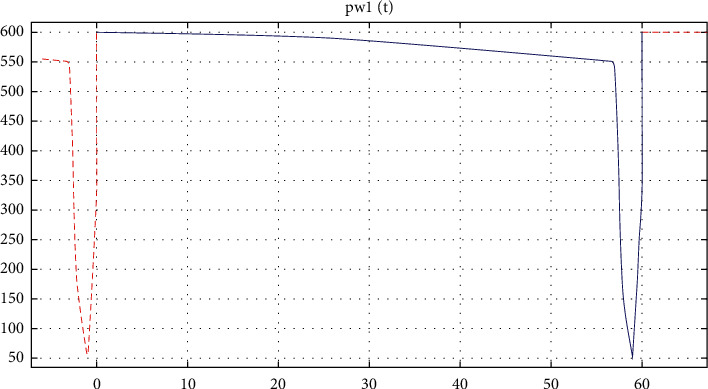
The temperature change of the moxa stick burning end in one minute.

Therefore, it is necessary to clean the ash regularly to ensure the radiation intensity. Moreover, the dust cannot be cleaned at the minimum temperature to ensure the therapeutic effect. In the simulation model, ash-cleaning was defined to once per minute. When cleaning, the moxa sticks were removed. Although there is no radioactive source, the air is heated when ash-cleaning. Therefore, the temperature of the radiative source was set to 50°C instead of 0°C when ash-cleaning. The given periodic temperature change is shown in Figure 3.

The moxa stick burning temperature, the moxa stick sizes, the stick-to-skin distance, and the ambient temperature are the primary parameters affecting the temperature distribution on the skin during mild moxibustion. The simulation models were used to investigate the influence of individual parameters on the mild moxibustion. Specifically, one parameter was adjusted while the other three were fixed. In practice, the maximum burning temperature of moxa sticks varies with the manufacturing process. When applying moxibustion, the doctor is selective about the stick-to-skin distance, and the manufacturer will also produce moxa sticks of different sizes according to different needs. When moxibustion is performed at different times, the ambient temperature will also change to a certain extent. The parameter values at various levels are listed in Table 2.

### 2.4. Simulation Verification

The simulation results were verified by the moxibustion experiment. The verification parameter is the average temperature of the stimulated area centered at the moxibustion point with a radius of 5 mm. The experimental data were taken from the moxibustion experiment at the Zusanli acupoint in the existing research [26]. The moxa stick diameter was 18 mm, the moxibustion time was 15 min, and the moxa stick burning end was 4 cm away from the skin surface. The moxa sticks were cleaned at the appropriate moments. The simulated and experimental values of temperature are compared in Table 3. The simulated temperature distribution roughly agrees with the experimental results. It proves that the proposed model and simulation results are reliable.

It can be seen from Table 3 that the difference between the two is less than 5%.

## 3. Results and Discussion

The factors affecting the effect of mild moxibustion are mainly external. The external factors mainly include the stick-to-skin distance, the moxa stick sizes, and the moxibustion time. Similarly, the year, leaf-to-velvet ratio, and density of moxa sticks influence the infrared spectral characteristics of moxibustion during combustion [33], but the year and the leaf-to-velvet ratio cannot be used as the parameters of the simulation model, and the simulation cannot be compared. The moxa sticks with different densities have little difference in infrared spectral intensity [33]. The density affects the stability and maximum temperature of combustion. Therefore, we comparatively analyzed the temperature curve near the maximum temperature when moxa sticks burn.

We first simulated the mild moxibustion under standard conditions and obtained the temperature curve under standard conditions. Then, the comparative experiments were performed for various parameters, such as the stick-to-skin distance, the moxa stick sizes, the burning temperature curve, and the moxibustion time. The experiments yielded a series of simulation results. The temperature rise curve of the moxibustion point and the temperature distribution on/inside the skin after the mild moxibustion were obtained from multiple sets of comparative experiments. This section analyzes the effect of each factor on heat penetration through an orthogonal experimental design. The findings are expected to help doctors to determine the moxibustion parameters faster and better in clinical practice.

### 3.1. Simulation Results Obtained under Standard Conditions

**Figure 4:**
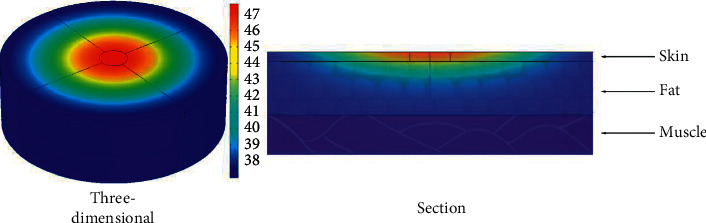
The temperature distribution of skin tissue after mild moxibustion under the standard condition.

The temperature distribution under various conditions is centered at the moxibustion point, and the temperature decreases in the surrounding direction. The temperature decays faster in the vertical direction than in the transverse direction. The shapes of the simulated temperature distribution are close in all conditions. The temperature distribution in the standard condition is shown in Figure 4.

**Figure 5:**
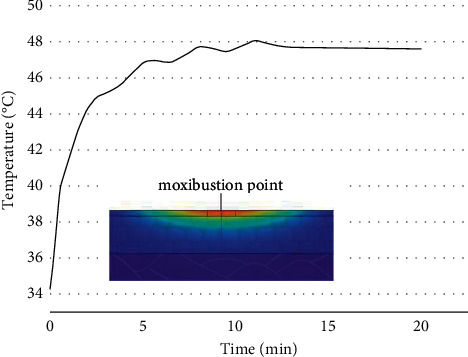
The temperature rise curve of moxibustion point under the standard condition.

**Figure 6:**
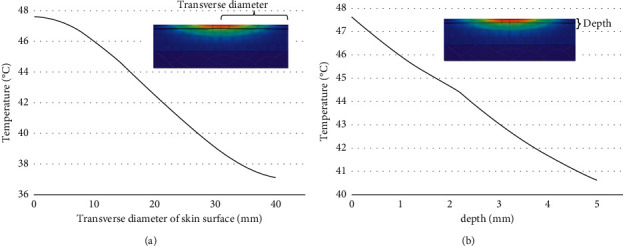
(a) The radial temperature curve on the skin surface under the standard condition (b) The vertical temperature curve at the moxibustion point under the standard condition.

The temperature rise curve is drawn based on the moxibustion point of the skin surface, as shown in Figure 5. The coordinates of the moxibustion point in the model are (0 mm, 0 mm, 25 mm). The simulation was set as the mild moxibustion in the standard condition. In this paper, the temperature of multiple points on the skin within 40 mm from the moxibustion point was extracted to obtain the temperature distribution of the skin surface after mild moxibustion. The temperature of multiple points within a vertical range of 5 mm from the moxibustion point was extracted to obtain the temperature distribution inside the skin tissue. The temperature distributions are shown in Figure 6.

Figure 5 shows that the temperature of the moxibustion point increases rapidly in the first half period. At about five minutes, the temperature fluctuates and the temperature rise slows down. In the subsequent 12-minute period, the temperature rise tends to be flat, reaching a stable temperature until the end of the mild moxibustion. Figure 6 shows that most areas on the skin surface can reach temperatures that are effective for stimulation. However, the depth with effective stimulation temperature is limited inside the skin tissue.

### 3.2. Influence of the Maximum Burning Temperature of the Moxa Stick on the Temperature Distribution

**Figure 7:**
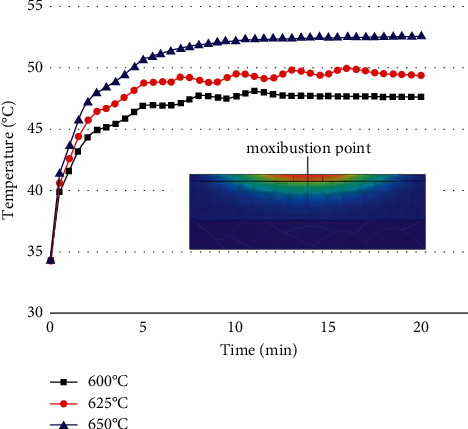
The temperature rise curve at the moxibustion point under the maximum burning temperature of various moxa sticks.

**Figure 8:**
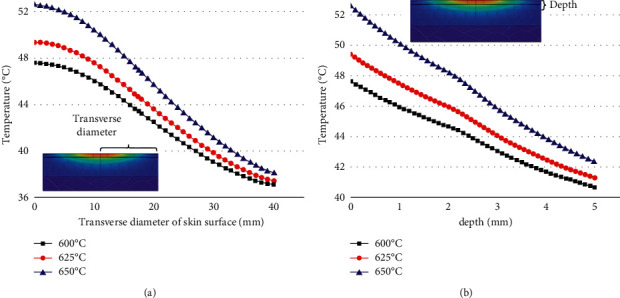
(a) The temperature rise curve in the transverse direction of the skin surface under the maximum burning temperature of various moxa sticks (b) The temperature rise curve in the vertical direction of the skin surface under the maximum burning temperature of various moxa sticks.

The maximum burning temperature of the moxa stick was changed under the condition that the moxibustion time, the stick-to-skin distance, and the moxa stick sizes were fixed. The findings include the temperature rise curve at the moxibustion point on the skin during the mild moxibustion and the temperature distribution on/inside the skin after the mild moxibustion, as shown in Figures 7 and 8.

Figures 7 and 8 suggest that the higher the burning temperature of moxa sticks, the higher the temperature of the skin surface after mild moxibustion, and the better the thermal penetration of mild moxibustion. However, as the burning temperature increases, the skin surface temperature after mild moxibustion may reach 60°C or even 70°C. It does not satisfy the optimum temperature requirements in the stimulation area, i.e., the interval of [46°C, 48°C].

### 3.3. Influence of Moxa Stick Sizes on Temperature Distribution

**Figure 9:**
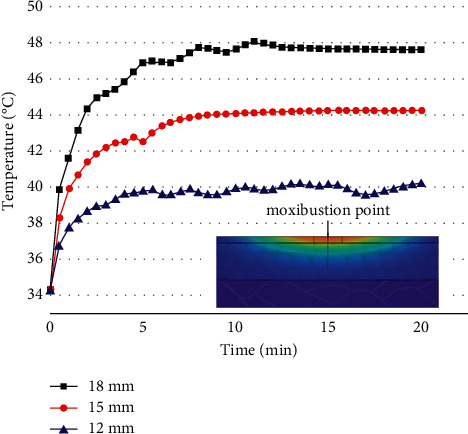
The temperature rise curve at the moxibustion point under various moxa stick sizes.

**Figure 10:**
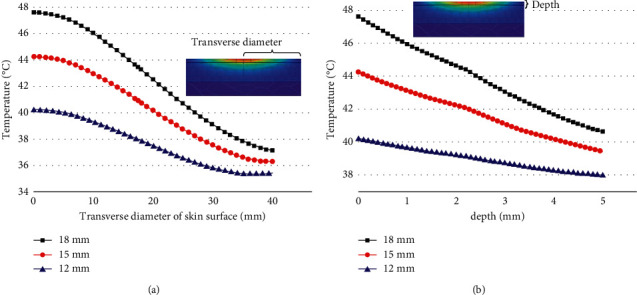
(a) The temperature rise curve in the transverse direction of the skin surface under various moxa stick sizes (b) The temperature rise curve in the vertical direction of the skin surface under various moxa stick sizes.

The moxa stick sizes were changed under the condition that the stick-to-skin distance, the moxibustion time, and the maximum burning temperature of the moxa stick were fixed. The findings include the temperature rise curve at the moxibustion point on the skin during the mild moxibustion and the temperature distribution on/inside the skin after the mild moxibustion, as shown in Figures 9 and 10.

Figures 9 and 10 present that the moxa stick sizes have a significant effect on the temperature distribution of the skin during moxibustion. Under the same conditions, the larger the moxa stick diameter, the higher the temperature at the moxibustion point, the faster the temperature rises, the higher the maximum burning temperature, and the better the thermal penetration of mild moxibustion.

### 3.4. Influence of Distance between Stick and Skin on Temperature Distribution

**Figure 11:**
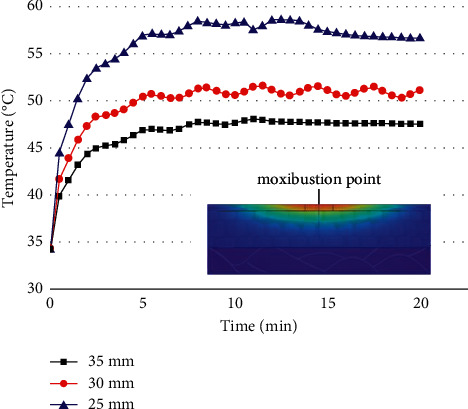
The temperature rise curve at the moxibustion point under different distances.

**Figure 12:**
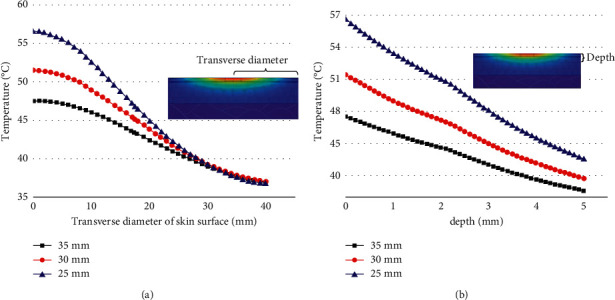
(a) The temperature rise curve in the transverse direction of the skin surface under different distances. (b) The temperature rise curve in the vertical direction of the skin surface under different distances.

The stick-to-skin distance was changed under the condition that the moxa stick sizes, the moxibustion time, and the maximum burning temperature of the moxa stick were fixed. The findings include the temperature rise curve at the moxibustion point on the skin during the mild moxibustion and the temperature distribution on/inside the skin after the mild moxibustion, as shown in Figures 11 and 12.

Figures 11 and 12 show that the closer the stick-to-skin distance, the higher the temperature at the moxibustion point, and the better the thermal penetration of mild moxibustion. However, the closer the distance, the faster the temperature drop along the transverse direction on the skin surface. And it has little effect on the temperature distribution inside the skin.

### 3.5. Influence of Ambient Temperature on Temperature Distribution

**Figure 13:**
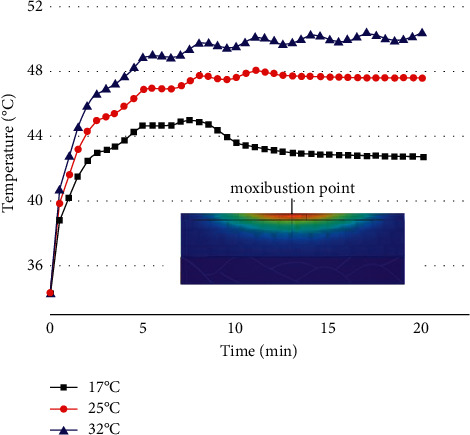
The temperature rise curve at the moxibustion point under different ambient temperatures.

**Figure 14:**
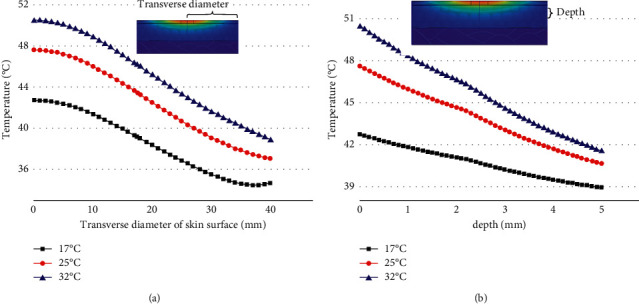
(a) The temperature rise curve in the transverse direction of the skin surface under different ambient temperatures. (b) The temperature rise curve in the vertical direction of the skin surface under different ambient temperatures.

The ambient temperature was changed under the condition that the moxa stick sizes, the stick-to-skin distance, and the maximum burning temperature of the moxa stick were fixed. The findings include the temperature rise curve at the moxibustion point on the skin during the mild moxibustion and the temperature distribution on/inside the skin after the mild moxibustion, as shown in Figures 13 and 14.

Figures 13 and 14 indicate that the higher the ambient temperature, the faster the temperature rises at the moxibustion point and the higher the maximum temperature. However, whether the ambient temperature is 17°C or 32°C, the temperature rise curve fluctuates wildly. There was even a drop in temperature at 17°C. The influence of ambient temperature on skin surface temperature distribution lies in the maximum temperature change after mild moxibustion. The shape of the temperature change curve is roughly the same. Also, the higher the ambient temperature, the higher the temperature inside the skin tissue.

### 3.6. Discussion

In this paper, the influence of various factors on the thermal penetration and the irritation to the skin surface in mild moxibustion was investigated. The temperature at a distance of 5 mm from the moxibustion point in the vertical direction was selected to measure the efficacy of mild moxibustion, analyze significant factors, and search for the optimal parameter combination. The coordinates of the reference point are (0 mm, 0 mm, 20 mm). The maximum burning temperature of moxa sticks, the moxa stick sizes, the stick-to-skin distance, and the ambient temperature were selected as the factors of the orthogonal experiment, expressed as *A*, *B*, *C*, and *D*. The number of factor levels is usually 2–4. We selected three levels for each factor, as listed in Table 4.

In mild moxibustion, three levels for each of the four factors were selected, written as *L*_9_(3^4^).

#### 3.6.1. Analysis of Orthogonal Experiment Results

Based on the mild moxibustion simulation model, the simulation analysis was performed on the nine groups of parameters in Table 5. The reference point temperature was used to compare the results, as listed in Table 6.

#### 3.6.2. Range Analysis

The range analysis (i.e., *R*-method) can be used to analyze the orthogonal experiment results for obtaining the optimal level combination and the influence of each factor on the experimental indicator. Specifically, a factor combination is obtained to optimize the experimental indicator, and all factors are sorted according to the degree of their influence on the experimental indicators.

The range analysis of heat penetration was implemented using the reference point temperature as the experimental indicator. The influence law of analysis of the influence of each parameter on the thermal penetration of moxibustion was revealed to obtain the best combination of operating parameters.

The findings in Table 7 present ${\bar{K}}_{A3}>{\bar{K}}_{A2}>{\bar{K}}_{A1}$, ${\bar{K}}_{B3}>{\bar{K}}_{B2}>{\bar{K}}_{B1}$, ${\bar{K}}_{C1}>{\bar{K}}_{C2}>{\bar{K}}_{C3}$, and ${\bar{K}}_{D3}>{\bar{K}}_{D2}>{\bar{K}}_{D1}$. It suggests that the combination of optimal parameter levels is A3B3C1D3. That is, when the burning temperature of the moxa stick is 650°C, the diameter of the moxa stick is 18 mm, the distance between the moxa stick and the skin is 25 mm, and the ambient temperature is 32°C; the thermal penetration of mild moxibustion is the best.

The *R*_*n*_ values of the parameters are sorted from largest to smallest as the moxa stick sizes, the stick-to-skin distance, ambient temperature, and the maximum burning temperature of the moxa stick. The parameter that has the most significant influence on the heat penetration is the moxa stick size, the second is stick-to-skin distance, and the smallest is the maximum burning temperature of the moxa stick.

#### 3.6.3. Analysis of Orthogonal Experimental Results Using the Variance Analysis

Due to the influence of various factors, the data obtained by the orthogonal experiment exhibit fluctuations. The factors that cause fluctuations can be divided into uncontrollable random factors and controllable changed factors in this study. The range analysis cannot distinguish between two types of factors. The variance analysis identifies the factors that have a significant impact on the experimental indicators based on the variance of the observed variables. It gives accurate quantitative estimation, as listed in Table 8.

The variance analysis shows that the most significant factor is the moxa stick size. The statistical significance of the ambient temperature and the stick-to-skin distance is close. And the significance of distance is slightly higher than that of ambient temperature, which is consistent with the range analysis results.

## 4. Conclusions

This paper creates the simulation model of mild moxibustion and does a single factor analysis. The contributions are summarized as follows.

First, the simulation model of mild moxibustion was established, and the simulation analysis under the standard condition of mild moxibustion was performed. The simulation and experimental results are analyzed comparatively. The difference between the simulation and experimental results was less than 5%. The comparison proves that the proposed model can accurately describe the temperature distribution of the mild moxibustion point in an actual situation.

Second, the univariate analysis was performed based on the four influencing factors, such as the maximum burning temperature of the moxa stick, the moxa stick sizes, the stick-to-skin distance, and the ambient temperature. By changing the level of one parameter and fixing other parameters, the temperature curve of the moxibustion point in mild moxibustion was drawn. According to the obtained curves, the influence characteristics of different parameters on the temperature distribution at the mild moxibustion point were revealed.

Third, the optimal design of the selected factors for the thermal penetration of mild moxibustion was carried out by orthogonal experiments. The orthogonal experiments were designed with the thermal penetration of mild moxibustion as the experimental indicator. The experimental findings suggest that the most influential factor on the thermal penetration of mild moxibustion is the moxa stick size, the second is the stick-to-skin distance, the third is the ambient temperature, and the smallest is the maximum burning temperature of the moxa stick. The influence of the parameters on the temperature distribution is of great significance to the choice of doctors in clinical treatment.

In this paper, in order to facilitate the simulation calculation, the thermophysical parameters of biological tissues are constants. However, in practical situations, the thermophysical parameters of biological tissues are often not constant. Therefore, in the follow-up work, the relationship between the thermophysical parameters of biological tissue and temperature will be considered. Besides, the selected parameters may involve uncertainties in actual situations. In the future, we will consider the effect of the uncertainties in the temperature distribution simulations to improve the clinical applicability of the proposed method.

## Figures and Tables

**Table 1:** The material parameters of biological tissues.

Biological tissue	*C*_*p*_(Jkg^−1^K^−1^)	*ρ*(kgm^−3^)	*k*(Wm^−1^K^−1^)
Skin	3391	1109	0.37
Fat	2348	911	0.21
Muscle	3421	1090	0.49

**Table 2:** The parameter values at various levels.

Levels	Maximum burning temperature of the moxa stick (°C)	Moxa stick sizes (mm)	Distance between stick and skin (mm)	Ambient temperature (°C)
1	600	12	25	17
2	650	15	30	25
3	700	18	35	32
Standard condition	600	18	35	25

**Table 3:** The comparison of simulated and experimental values of temperature.

Time (min)	Simulated temperature (°C)	Test temperature (°C)	Deviation (%)
0	34.30	34.3 ± 0.4	0
0.5	38.56	40.0 ± 2.0	−3.73
1	40.03	41.4 ± 2.4	−3.42
1.5	41.29	42.8 ± 2.7	−1.87
2	42.25	43.0 ± 3.0	3.66
2.5	42.66	43.0 ± 2.9	−0.80
3	43.05	43.0 ± 3.1	0.12
3.5	43.30	43.0 ± 3.0	0.70
4	43.18	44.0 ± 2.3	−1.90
4.5	43.06	44.1 ± 2.3	−2.42
5	43.07	44.6 ± 2.9	−3.55
5.5	43.42	44.1 ± 3.0	−1.57
6	43.77	44.2 ± 2.7	−0.98
6.5	43.97	44.2 ± 2.5	−0.52
7	43.75	44.4 ± 2.2	−1.49
7.5	43.54	44.6 ± 2.1	−2.48
8	43.46	44.5 ± 2.4	−2.39
8.5	43.75	44.7 ± 2.6	−2.17
9	44.05	44.9 ± 2.3	−1.93
9.5	44.20	44.7 ± 1.9	−1.13
10	43.95	44.2 ± 1.5	−0.57
10.5	43.70	44.2 ± 2.3	−1.14
11	43.59	43.9 ± 2.5	−0.71
11.5	43.87	44.5 ± 2.2	−1.44
12	44.14	44.1 ± 1.6	0.01
12.5	44.27	44.5 ± 1.7	−0.52
13	44.02	44.0 ± 1.6	0.00
13.5	43.76	43.4 ± 1.7	0.83
14	43.64	43.1 ± 1.6	1.25
14.5	43.91	42.6 ± 1.1	3.08
15	44.18	43.1 ± 1.8	2.51

**Table 4:** The level of each factor.

Levels	Maximum burning temperature of the moxa stick (*A*) (°C)	Moxa stick sizes (*B*) (°C)	Distance between stick and skin (*C*) (mm)	Ambient temperature (*D*) (mm)
1	600	12	25	17
2	625	15	30	25
3	650	18	35	32

**Table 5:** The standard orthogonal table of *L*_9_(3^4^).

Experimental no.	Maximum burning temperature of the moxa stick (*A*)	Moxa stick sizes (*B*)	Distance between stick and skin (*C*)	Ambient temperature (*D*)
1	600	12	25	17
2	600	15	30	25
3	600	18	35	32
4	625	12	30	32
5	625	15	35	17
6	625	18	25	25
7	650	12	35	25
8	650	15	25	32
9	650	18	30	17

**Table 6:** The orthogonal experimental results of mild moxibustion.

Experimental no.	Maximum burning temperature of the moxa stick (*A*)	Moxa stick sizes (*B*)	Distance between stick and skin (*C*)	Ambient temperature (*D*)	(0,0,20 mm) temperature
1	600	12	25	17	38.782
2	600	15	30	25	40.533
3	600	18	35	32	41.547
4	625	12	30	32	40.146
5	625	15	35	17	38.370
6	625	18	25	25	43.483
7	650	12	35	25	38.328
8	650	15	25	32	43.615
9	650	18	30	17	41.379

**Table 7:** The temperature range analysis at the reference point of (0 mm, 0 mm, 20 mm).

Experimental no.	Maximum burning temperature of the moxa stick (*A*)	Moxa stick sizes (*B*)	Distance between stick and skin (*C*)	Ambient temperature (*D*)	(0,0,20 mm) temperature
1	600	12	25	17	38.782
2	600	15	30	25	40.533
3	600	18	35	32	41.547
4	625	12	30	32	40.150
5	625	15	35	17	38.856
6	625	18	25	25	43.713
7	650	12	35	25	38.730
8	650	15	25	32	44.727
9	650	18	30	17	41.835
*K*_*n*1_	40.29	39.22	42.41	39.82	
*K*_*n*2_	40.91	41.37	40.84	40.99	
*K*_*n*3_	41.76	42.37	39.71	42.14	
${\bar{K}}_{n1}$	13.43	13.07	14.14	13.27	
${\bar{K}}_{n2}$	13.64	13.79	13.61	13.66	
${\bar{K}}_{n3}$	13.92	14.12	13.24	14.05	
Optimal levels	A3	B3	C1	D3	
*R*_*n*_	1.48	3.14	2.7	2.32	

**Table 8:** The temperature variance analysis at the reference point of (0 mm, 0 mm, 20 mm).

Variance source	*f*_*i*_	*F*	Significance
Maximum burning temperature of the moxa stick	2	0.29	
Moxa stick sizes	2	2.08	^*∗∗∗*^
Distance between stick and skin	2	1.23	^*∗∗*^
Ambient temperature	2	0.81	^*∗∗*^

## Data Availability

Data sharing is not applicable to this article as no new data were created or analyzed in this study.
